# Outcomes and complication rates of different bone grafting modalities in long bone fracture nonunions: a retrospective cohort study in 182 patients

**DOI:** 10.1186/1749-799X-8-33

**Published:** 2013-09-09

**Authors:** Michael A Flierl, Wade R Smith, Cyril Mauffrey, Kaan Irgit, Allison E Williams, Erin Ross, Gabrielle Peacher, David J Hak, Philip F Stahel

**Affiliations:** 1 Department of Orthopaedic Surgery, Denver Health Medical Center, University of Colorado Denver, School of Medicine, 777 Bannock Street, Denver, CO 80204, USA; 2 Department of Orthopaedic Surgery, Geisinger Medical Center, 100 North Academy Ave, Danville, PA 17825, USA; 3 Bay Pines VA Healthcare System, Bay Pines, St, Petersburg, FL 33744, USA

**Keywords:** Fracture nonunion, Autograft, Allograft, Bone morphogenetic protein

## Abstract

**Background:**

Novel bone substitutes have challenged the notion of autologous bone grafting as the ‘gold standard’ for the surgical treatment of fracture nonunions. The present study was designed to test the hypothesis that autologous bone grafting is equivalent to other bone grafting modalities in the management of fracture nonunions of the long bones.

**Methods:**

A retrospective review of patients with fracture nonunions included in two prospective databases was performed at two US level 1 trauma centers from January 1, 1998 (center 1) or January 1, 2004 (center 2), respectively, until December 31, 2010 (*n* = 574). Of these, 182 patients required adjunctive bone grafting and were stratified into the following cohorts: autograft (*n* = 105), allograft (*n* = 38), allograft and autograft combined (*n* = 16), and recombinant human bone morphogenetic protein-2 (rhBMP-2) with or without adjunctive bone grafting (*n* = 23). The primary outcome parameter was time to union. Secondary outcome parameters consisted of complication rates and the rate of revision procedures and revision bone grafting.

**Results:**

The autograft cohort had a statistically significant shorter time to union (198 ± 172–225 days) compared to allograft (416 ± 290–543 days) and exhibited a trend towards earlier union when compared to allograft/autograft combined (389 ± 159–619 days) or rhBMP-2 (217 ± 158–277 days). Furthermore, the autograft cohort had the lowest rate of surgical revisions (17%) and revision bone grafting (9%), compared to allograft (47% and 32%), allograft/autograft combined (25% and 31%), or rhBMP-2 (27% and 17%). The overall new-onset postoperative infection rate was significantly lower in the autograft group (12.4%), compared to the allograft cohort (26.3%) (*P* < 0.05).

**Conclusion:**

Autologous bone grafting appears to represent the bone grafting modality of choice with regard to safety and efficiency in the surgical management of long bone fracture nonunions.

## Introduction

Fracture nonunions of long bones continue to represent a significant clinical challenge and socioeconomic burden, associated with high complication rates and the potential for poor long-term outcomes [1-5]. Autologous bone grafting has received a negative reputation in the past, mainly due to the high risk of postoperative complications related to the harvesting procedure [6]. However, more recent innovative and minimally invasive harvesting techniques have mitigated the historic issue of donor site morbidity and renewed the interest in autologous bone as a preferred source for bone grafting [7-9].

The introduction of new generation bone substitutes and recombinant molecules with osteoinductive properties has recently challenged the role of autologous bone grafting as the ‘gold standard’ for the surgical treatment of nonunions [10-12]. In light of the immense market potential for bone graft substitutes and related products, estimated to be US$1 billion in the USA alone, the push for new ‘osteobiologicals’ may in large part be industry-driven, rather than based on objective patient safety and quality data [13-15]. In fact, well-publicized concerns have recently been raised regarding the safety of the uncritical application of recombinant bone morphogenetic protein (BMP)-2 for a variety of indications, including its off-label use [14,16-18].

The present study was designed to determine the ‘ideal’ modality of adjunctive bone grafting in the management of long bone fracture nonunions. We hypothesized that the use of autograft is equivalent to other bone grafting options with regard to healing times and complication rates.

## Methods

### Study design

**Figure 1:**
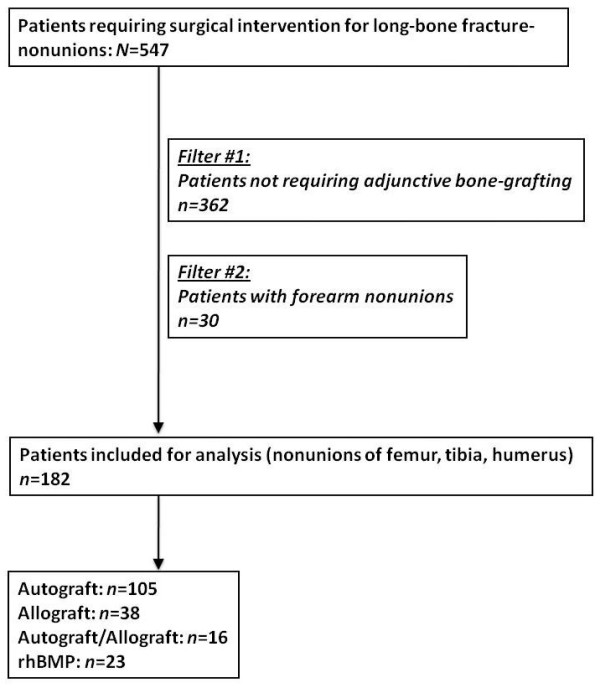
Patient selection flowchart.

**Figure 2:**
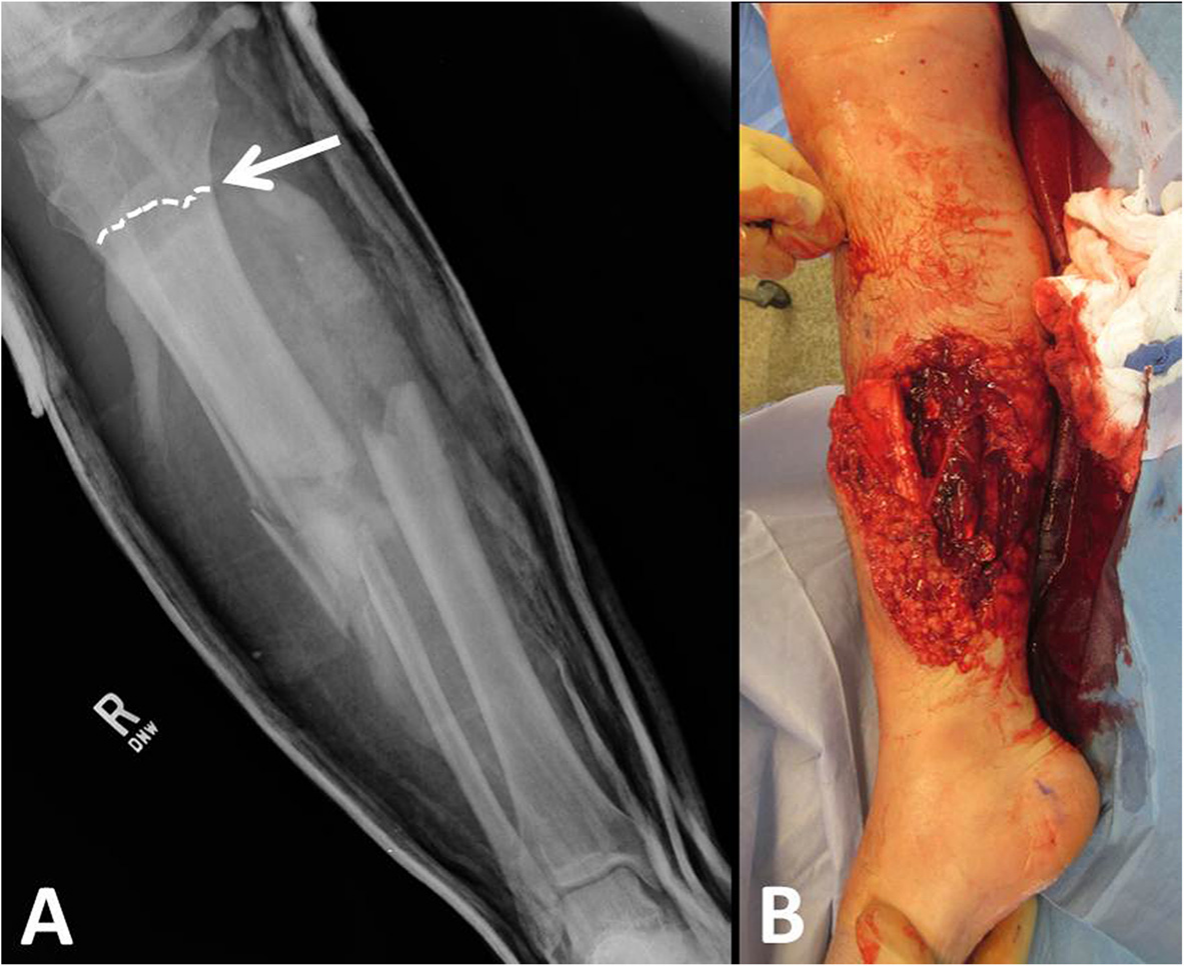
**Representative case example.** A 31-year-old male pedestrian was hit at high speed by a motor vehicle, sustaining an isolated type IIIB open segmental tibia shaft fracture **(A**, **B)** with associated segmental fibula fracture (AO/OTA type 42-C2.2). The arrow in panel **A** depicts the proximal nondisplaced fracture line of the segmental tibia fracture.

**Figure 3:**
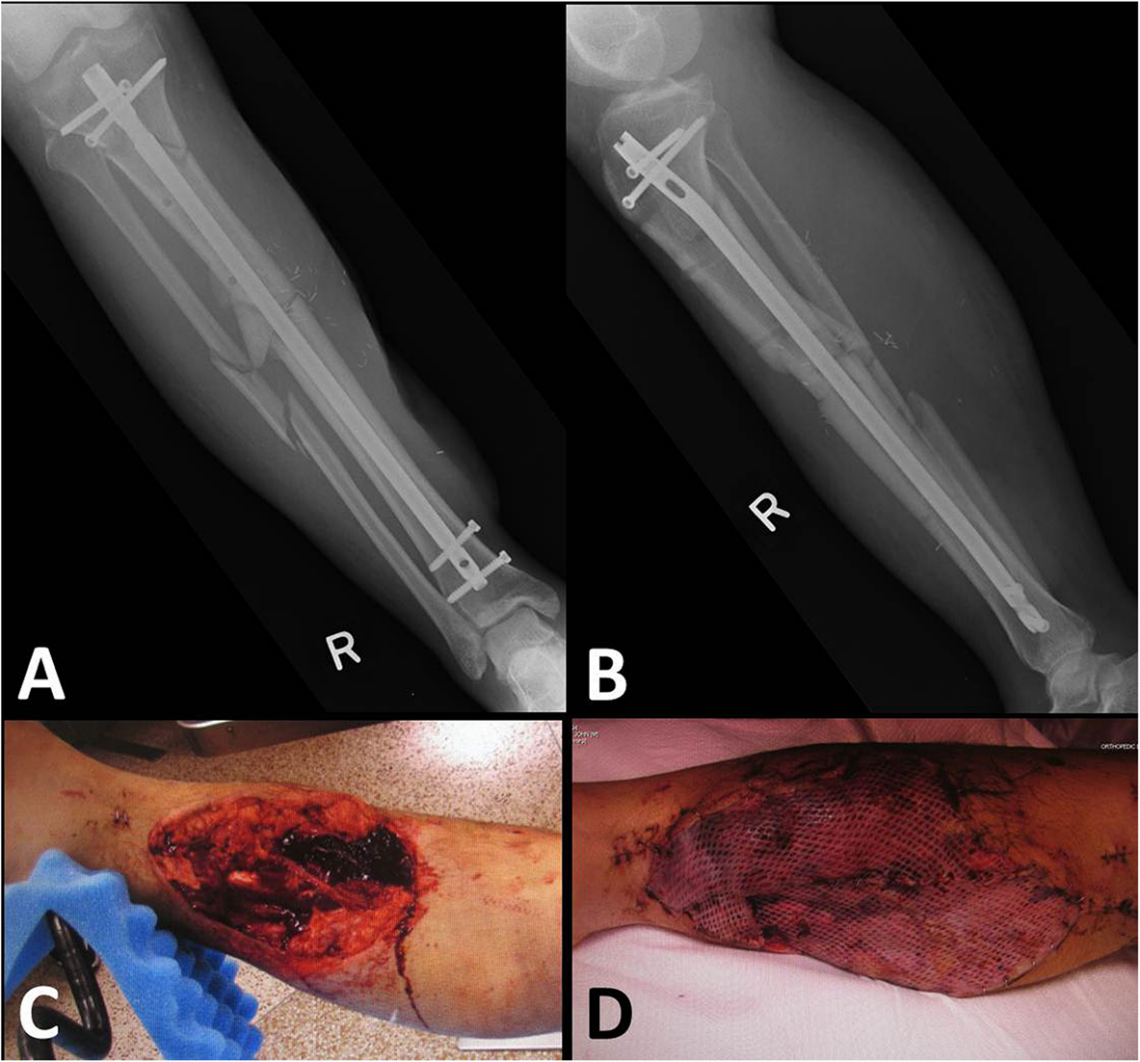
**Identical case as in Figure**2**.** After initial spanning external fixation and surgical debridement, the patient was taken back to the operating room the next day for definitive surgical management by reamed intramedullary interlocking nail fixation **(A**, **B)**, repeat surgical debridement **(C)**, and soft tissue coverage by free microvascular gracilis flap in the same session **(D)**.

**Figure 4:**
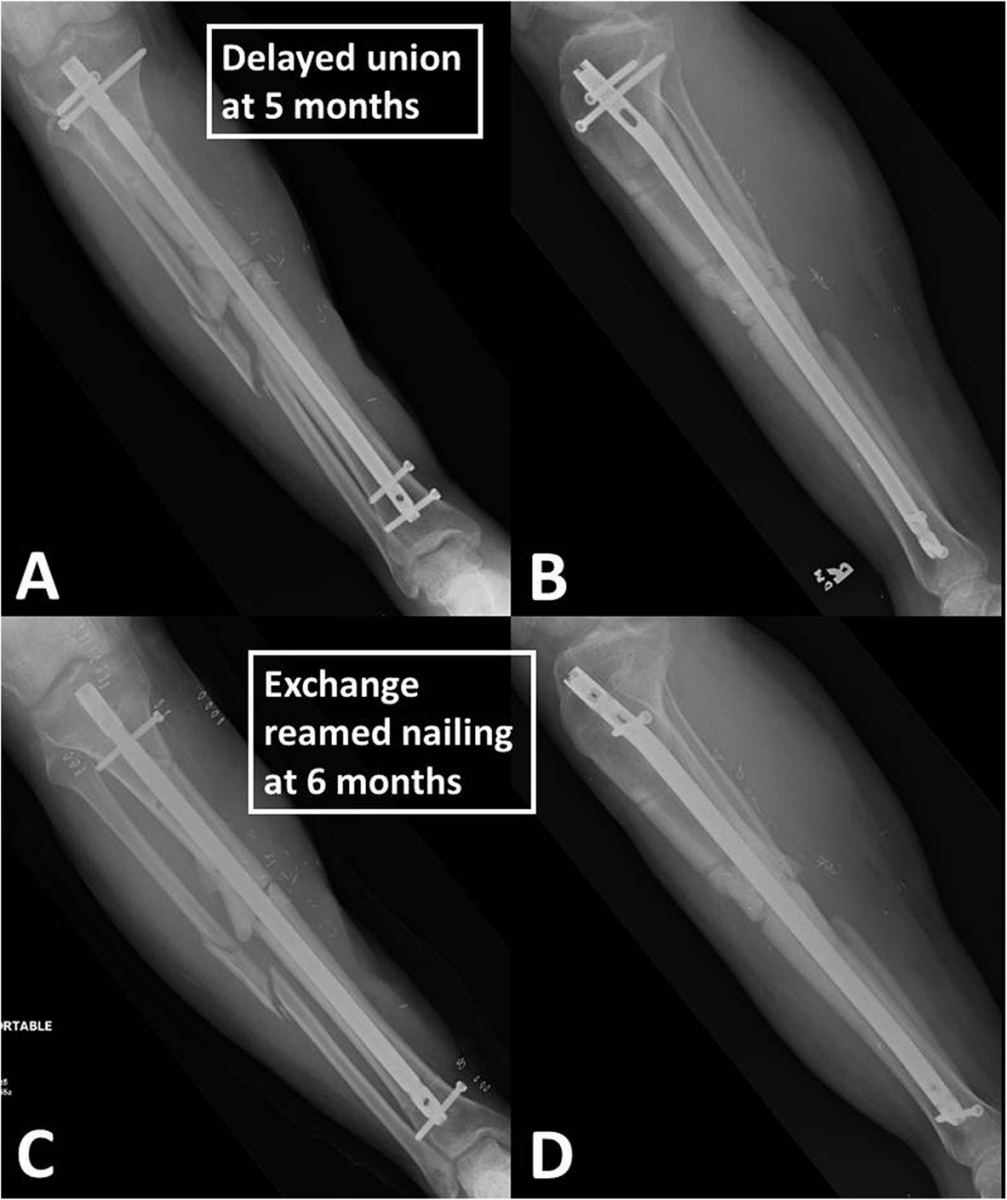
**Identical case as in Figures**2**,**3**.** At 5 months follow-up, the patient showed clinical and radiographic signs of a nonunion of the open tibia fracture **(A**, **B)**. A surgical revision was performed by exchange reamed nailing at 6 months **(C**, **D)**.

**Figure 5:**
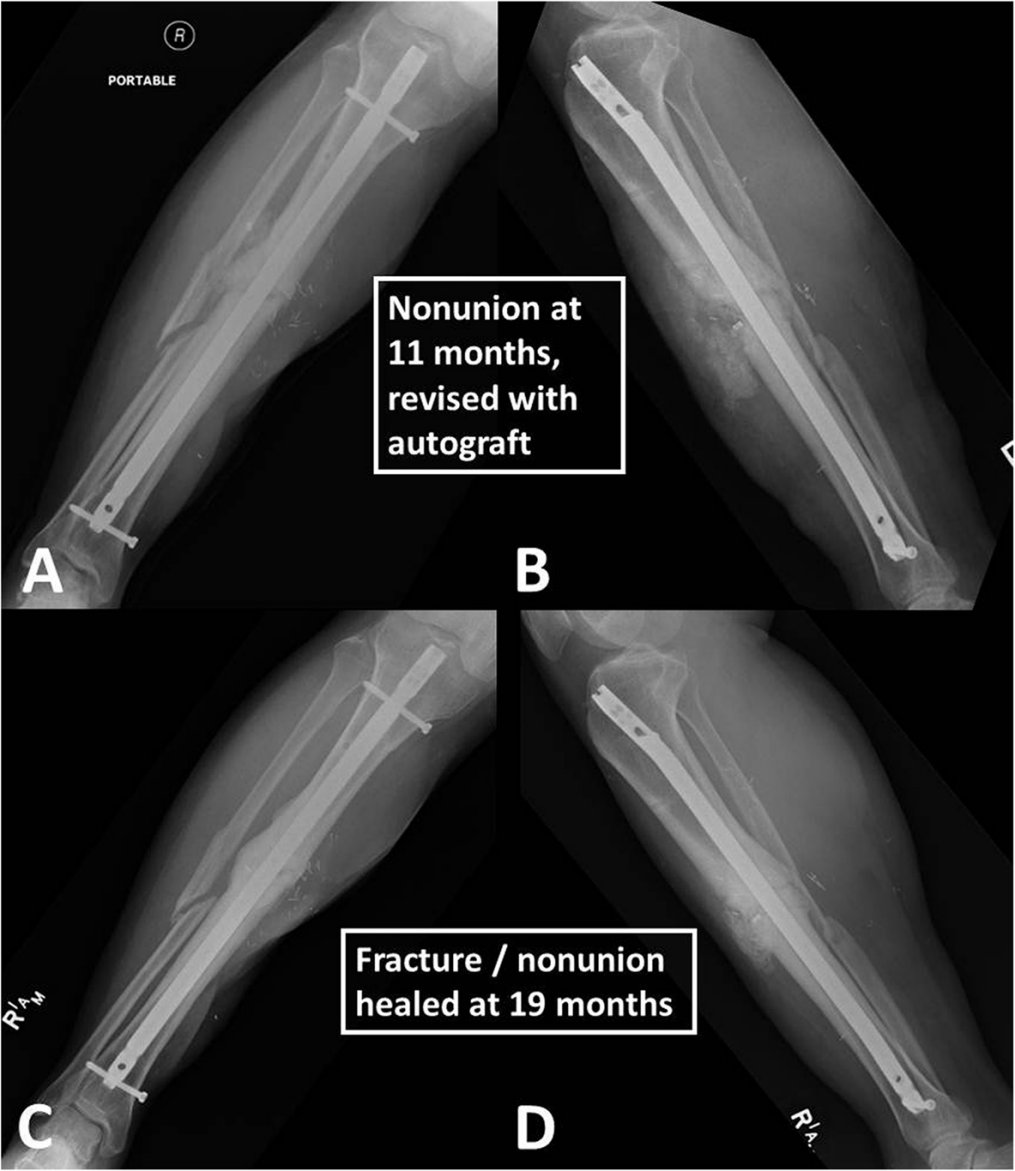
**Identical case as in Figures**2**,**3**,**4**.** The nonunion persisted at 11 months post injury, and a second revision was performed by decortication and application of a central bone graft **(A**, **B)**, using a reamer-irrigator-aspirator technique for minimal invasive autograft harvest from the ipsilateral femur. The fracture/nonunion was clinically and radiographically healed at 19 months post injury **(C**, **D)**, and the patient was asymptomatic with a full functional recovery.

A retrospective cohort study was designed based on two prospective institutional databases from representative level 1 trauma centers in the USA (Denver Health Medical Center, Denver, CO; and Geisinger Medical Center, Danville, PA). Prior to study initiation, approval by the two respective Institutional Review Boards was obtained. The databases included all patients between 18 and 85 years of age, who were admitted to one of the two participating centers for surgical treatment of long bone fracture nonunions between January 1, 1998 and December 31, 2010 (*n* = 373; Denver Health), and between January 1, 2004 and December 31, 2010 (*n* = 201; Geisinger), respectively. Patients that did not require adjunctive bone grafting (*n* = 362) or had a nonunion of the forearm (*n* = 30) were excluded from the analysis. Patients who were lost to follow-up prior to a minimum of 12 months postoperatively or prior to clinical and radiographic union were excluded. The remaining 182 patients who required adjunctive bone grafting for nonunions of the tibia (*n* = 98), femur (*n* = 46), or humerus (*n* = 38) were included for final analysis. The flowchart in Figure 1 depicts the patient selection process in detail. Figures 2,3,4,5 illustrate a representative case example of a 31-year-old male who sustained an isolated type IIIB open segmental tibia fracture. The patient underwent initial surgical debridement and external fixation, followed by staged conversion to an intramedullary nail. He developed a tibial nonunion, requiring exchange nailing and autologous bone grafting.

In accordance with the definition by the US Federal Drug Administration (FDA), nonunion was defined as a fractured bone which had not completely healed within 9 months of injury and which had not shown signs of progression towards healing for three consecutive months on serial radiographs [19]. Nonunions were classified according to the Weber and Cech classification system into hypertrophic, normotrophic, or atrophic [19]. Nonunions were treated either by plate osteosynthesis (using conventional plates or locking plate systems), intramedullary nail fixation (including exchange nailing), or by external ring fixator in combination with bone grafting, based on the individual surgeon’s preference.

Patients were stratified into the following cohorts for analysis, based on the bone grafting modality: (1) autograft (*n* = 105), (2) allograft (*n* = 38), (3) allograft in combination with autograft (*n* = 16), and (4) rhBMP-2 with or without adjunctive bone grafting substitute (*n* = 23). The rhBMP-2 product was mixed with sterile saline and prepared immediately prior to use from a kit containing all necessary components, according to the manufacturer’s instructions (Infuse®, Medtronic, Memphis, TN, USA). In 6 patients, rhBMP-2 was administered without bone grafting adjunct, while in the remaining 17 patients rhBMP-2 was combined with allograft. All patients were followed postoperatively for a minimum of 12 months, or until clinical and radiographic bone healing occurred. ‘Clinical union’ was defined as painless weight bearing and lack of local tenderness over the nonunion site on physical examination [20,21]. ‘Radiographic union’ was defined as bridging bone on a minimum of three cortices on conventional antero-posterior and lateral radiographs. The application of rhBMP-2 for the revision of long bone fracture nonunions was considered an ‘off-label’ indication. Autograft donor sites were as follows: (1) iliac crest (*n* = 64), (2) femoral reamer-irrigator-aspirator (*n* = 20), and (3) other (*n* = 10). The primary outcome parameter was time to union. The secondary outcome parameters included the incidence of new-onset postoperative infection in a previously noninfected nonunion or ongoing infection in infected nonunions, as well as the need of revision surgeries and revision bone grafting.

### Statistical analysis

Data were analyzed using SPSS software version 17.0 (SPSS Inc., Chicago, IL, USA). Descriptive statistics were performed to summarize demographic and clinical variables and to evaluate distributional characteristics of continuous variables, using Kruskal-Wallis test, Mann–Whitney U test, and Chi-square test, as appropriate. Data are expressed as means ± standard deviation or means ± 95% confidence interval (95% CI), as deemed appropriate. Statistical significance was defined at *P* < 0.05.

## Results

### Patient demographics

A total of 182 patients (102 males and 80 females) met the inclusion criteria (Figure 1). The mean age was 44 ± 13.6 years. Sixty-eight patients were confirmed smokers, and 30 patients had a history of smoking and reported cessation prior to nonunion surgery. The remaining 84 patients reported to have never smoked. Demographic data, stratified by subgroups, are depicted in Table 1. The surgical management of the nonunions consisted of plate osteosynthesis (65%), intramedullary interlocking nail fixation (28%), or application of an external ring fixator (7%). Nonunion characteristics and management strategies in the different cohorts are shown in Table 2.

**Table 1:** Patient demographics and fracture characteristics

**Variable**	**Overall**	**Autograft**	**Allograft**	**Autograft/allograft**	**rhBMP-2**
Gender					
Male	102 (56%)	58 (55%)^*^	16 (42%)	4 (25%)	7 (30%)
Female	80 (44%)	47 (45%)^*^	22 (58%)	12 (75%)	16 (70%)
Mean age (years)	44 (±13.6)	41 (±12.5)^*^	47 (±13)	46 (±14)	52 (±15)
EtOH					
Abuse	44 (24%)	22 (21%)	8 (21%)	0	5 (22%)
Occasionally	45 (25%)	30 (29%)	12 (32%)	6 (37%)	6 (26%)
Never	97 (51%)	53 (50%)	18 (47%)	10 (63%)	12 (52%)
Tobacco smoking					
Current	68 (37%)	41 (39%)	16 (42%)	4 (25%)	6 (26%)
Former	30 (17%)	17 (16%)	4 (11%)	4 (25%)	5 (22%)
Never	84 (46%)	47 (45%)	18 (47%)	8 (50%)	12 (52%)
Illicit drug use					
Current	28 (15%)	17 (16%)	9 (24%)	0**	1 (4%)
Former	7 (4%)	6 (6%)	0	0**	1 (4%)
Never	147 (81%)	82 (78%)	29 (76%)	16 (100%)	21 (92%)
Injury mechanism					
MVA/MCA	83 (46%)	49 (47%)	18 (47%)	8 (50%)	8 (35%)
Fall	39 (21%)	20 (19%)	10 (26%)	3 (19%)	6 (26%)
GSF	6 (3%)	5 (4%)	1 (3%)	0	0
Other	33 (18%)	31 (30%)	9 (24%)	5 (31%)	9 (39%)
Fracture location					
Tibia	98 (54%)	57 (54%)	21 (55%)	9 (56%)	11 (48%)
Femur	46 (25%)	28 (27%)	9 (24%)	4 (25%)	5 (22%)
Humerus	38 (21%)	20 (19%)	8 (21%)	3 (19%)	7 (30%)
Fracture characteristics					
Open	79 (43%)	51 (49%)	17 (45%)	4 (25%)	7 (30%)
Closed	103 (57%)	54 (51%)	21 (55%)	12 (75%)	16 (70%)

*MVA* motor vehicle accident, *MCA* motorcycle accident, *GSF* gunshot fracture, *rhBMP*-2 recombinant human bone morphogenetic protein-2.

*(*P* < 0.05), statistically significant difference among autograft cohort vs. allograft, autograft/allograft, and BMP-2 cohorts.

** (*P* < 0.05) statistically significant difference between autograft/allograft group and remaining cohorts.

**Table 2:** Nonunion characteristics and management strategies

**Variable**	**Overall (%)**	**Autograft (%)**	**Allograft (%)**	**Allograft/autograft (%)**	**rhBMP-2 (%)**
Nonunion classification					
Atrophic	55 (30)	35 (34)	11 (29)	4 (25)	6 (26)
Normotrophic	75 (41)	44 (42)	16 (43)	7 (44)	9 (39)
Hypertrophic	21 (12)	13 (12)	4 (10)	2 (12)	2 (9)
Infected	31 (17)	13 (12)	7 (18)	3 (19)	6 (26)
Initial surgical fracture management					
Plate	119 (65)	79 (75)	22 (58)	6 (38)	13 (57)
IMN	50 (28)	19 (18)	13 (34)	8 (50)	9 (39)
Ring fixator	13 (7)	7 (7)	3 (8)	2 (12)	1 (4)

*IMN* intramedullary nail, *rhBMP*-2 recombinant human bone morphogenetic protein-2.

Patients in the autograft group had a significantly shorter average time to union (198 days, 95% CI 172–225 days), when compared to the allograft group (416 days, 95% CI 290–543 days), while no statistically significant differences were found among the autograft group, the autograft/allograft cohort (389 days, 95% CI 159–619 days), and the rhBMP-2 cohort (217 days, 95% CI 158–277 days). Differences in time to healing were significant between the autograft and allograft cohorts (*P* < 0.001).

Furthermore, the autograft group had a significantly lower incidence of surgical revision rates (17.1%) and need for revision bone grafting (8.6%) compared to the allograft group (47.4% and 31.6%, respectively), the allograft/autograft group (25.0% and 31.3%, respectively), and the rhBMP-2 cohort (26.1% and 17.4%, respectively) (*P* < 0.01 for autograft vs. all other cohorts). Stratified by subgroups, the incidence of new-onset postoperative infection after nonunion surgery was highest in the allograft group (26.3%), followed by the rhBMP-2 group (17.4%), autograft group (12.4%), and was found to be lowest in the autograft/allograft cohort (6.3%). These data were statistically significant between autograft and allograft cohorts (*P* < 0.05) and were further stratified by superficial and deep infection. A summary of the outcome data is shown in Table 3.

**Table 3:** Outcome parameters

**Variable**	**Autograft**	**Allograft**	**Autograft/allograft**	**rhBMP-2**
*n*	105	38	16	23
Time to union in days (mean ± 95% CI)	198* (172–225)	416 (290–543)	389 (159–619)	217 (158–277)
Surgical revision rate (any unplanned return to the OR)	18** (17.1%)	18 (47.4%)	4 (25.0%)	6 (26.1%)
Revision bone grafting/nonunion rate	9** (8.6%)	12 (31.6%)	5 (31.3%)	4 (17.4%)
New-onset postoperative infection	13*** (12.4%)	10 (26.3%)	1 (6.3%)	4 (17.4%)
Superficial	5 (4.8%)	1 (2.6%)	0 (0%)	0 (0%)
Deep	8 (7.6%)	9 (23.7%)	1 (6.3%)	4 (17.4%)
Persisting infection	3 (2.9%)	3 (7.9%)	1 (6.3%)	1 (4.3%)

*rhBMP*-2 recombinant human bone morphogenetic protein-2.

Data in the time to union row are expressed as mean ± 95% CI.

* (*P* < 0.001), statistically significant between autograft and allograft cohorts.

** (*P* < 0.01), statistically significant between autograft and all other cohorts.

*** (*P* < 0.05), statistically significant between autograft and allograft groups.

## Discussion

The present study was designed to analyze the efficacy of distinct adjunctive bone grafting modalities for surgical revisions of long bone fracture nonunions. Our data revealed that in this particular patient population, the use of autograft had a significantly shorter time to union when compared to allograft, the lowest incidence of surgical revision rates and revision bone grafting, and the lowest postoperative infection rates, compared to the allograft alone, allograft/autograft or rhBMP-2 comparative cohorts. There was a statistically nonsignificant trend towards earlier time to union in autograft patients compared to autograft/allograft or rhBMP-2 cohorts. These findings support the notion that autograft remains the gold standard for bone grafting of nonunions [22], despite the emerging availability of a multiplicity of novel osteobiologicals that have recently received considerable attention as bone grafting adjuncts [23]. The indication spectrum of human recombinant rhBMP-2 remains highly controversial, particularly in light of some recent concerns regarding its questionable safety and efficiency profile [14,16-18]. The historic ‘rhBMP-2 Evaluation in Surgery for Tibial Trauma (BESTT)’ multicenter prospective randomized trial on 450 patients with open tibial fractures managed by unreamed interlocking nail fixation revealed that the local application of rhBMP-2 resulted in a significant reduction of nonunion rates [24]. Jones et al. reported the results from a randomized, controlled clinical trial on 30 patients, which revealed that rhBMP-2 in combination with allograft had similar times to union and outcomes compared to autograft in tibial shaft fractures with cortical defects [25]. A more recent prospective, randomized trial failed to demonstrate accelerated healing in open tibia fractures treated by reamed intramedullary nailing and rhBMP-2, confirming the notion that reaming represents a more crucial parameter for successful fracture healing, rather than the adjunctive use of rhBMP-2 [26].

Our present study supports the notion that autograft remains at least as effective as rhBMP-2, with similar time to union and decreased incidence of postoperative complications and requirement for surgical revisions. These findings are challenged by a different recent publications, which compared rhBMP-2 with cancellous allograft and iliac crest autograft in a retrospective study of 89 patients with long bone nonunions [27].The authors failed to find a difference in union rates between the treatment groups and reported an increased incidence of postoperative infections in the autologous iliac crest bone grafting group, compared to the rhBMP-2/allograft cohort [27]. In addition, iliac crest autograft was associated with longer operative procedures and a higher amount of intraoperative blood loss. The authors concluded that rhBMP-2 may provide a viable alternative to autologous iliac bone grafting in the management of long bone nonunions [27].

The lack of long-term safety and efficiency data on the use of BMPs as bone grafting adjuncts, in conjunction with realistic concerns that BMPs may be associated with the long-term induction of bone tumors [28,29], substantiates the ongoing discussion against liberal application of BMPs until long-term data are available [16,30]. Ritting et al. recently published a pediatric case report describing a massive local inflammatory reaction after use of rhBMP-2 for repair of a symptomatic forearm nonunion in a child [31]. Such serious complications question the standard use of rhBMP-2 as routine bone grafting adjuncts in nonunion surgery.

Demineralized bone matrix (DBM) represents the most commonly used bone substitute with osteoinductive properties [32]. With the use of DBM, however, an adjunctive scaffold is needed to add osteoconductive properties [32]. Comparison of combined osteogenic products have demonstrated significant differences in osteogenic potential, depending on processing techniques, carriers, sterilization methods, and storage conditions [33,34]. Such variations result in considerable inconsistency in product safety and efficiency. Bae and colleagues recently reported substantial ‘lot-to-lot’ variability in terms of BMP concentrations and *in vivo* fusion rates in DBM/BMP products, challenging the reliability of such adjuncts in bone grafting [35,36].

Autograft possesses the combined osteogenic properties of osteoinduction and osteoconduction, thus representing superior mechanical and biologic properties than any allogeneic or synthetic bone substitutes [37]. Traditionally, autograft was harvested from the iliac crest, providing sizeable quantities of autograft and the possibility of a tricortical structural grafting [38]. However, disadvantages of using the iliac crest as a donor site are well established in the literature and include persistent pain, infections, and the induction of iatrogenic pelvic fractures and nonunions [6,39,40].

An emerging alternative to iliac crest harvesting is represented by the Reamer-Irrigator-Aspirator (RIA) system, which allows the harvest of significant amounts of autograft from the intramedullary femoral canal [7,41].

Compared to iliac crest harvest, the RIA produces comparable volumes of bone graft and comparable harvesting times, but with a markedly reduced incidence of postoperative pain and lower rates of postoperative complications [42]. The grafting material obtained by RIA possesses osteogenic properties and, although morselized, appears to provide three-dimensional properties similar to trabecular bone and offers the presence of osteoinductive BMPs and growth factors in the grafting material [43,44].

Our study has several limitations. First, the retrospective nature of the study design is associated with the known pitfalls of any retrospective analysis and thus limits the scientific value of the conclusions. Secondly, our study includes data from two different level 1 trauma centers, adding not only increased surgeon variability, but also resulting in significant differences in the modality of data acquisition between the study sites. On the other hand, this weakness may, at the same time, represent a strength, since the inclusion of two different study sites may adjust for confounding variables specific to a single institution, and thus make the findings more generally applicable. Another drawback of our study is the combined analysis of three different long bones with unequal biomechanical and biological properties with regard to the exposure to axial loading (femur, tibia) vs. rotational forces (humerus), quality of the surrounding soft tissue envelope and blood flow to the fracture site, etc. However, when attempting a subanalysis for individual long bones (humerus vs. femur vs. tibia), the sample sizes and power became too small to make reasonable statistical assumptions without the risk of a type-II error. In line, while differences exist between gender distribution and age between cohorts, subanalyses rendered patient numbers and power too small to extract meaningful statistical data without significant risk for a type-II error. Moreover, the incidence of surgical revision in the autograft group may have been reduced as these patients have received the current gold standard treatment. Finally, there is the possibility of selection bias in the present study. As bone grafting modality was left to the treating surgeon’s discretion, it is possible that patients were treated differently based upon various pre-existing patient factors. As this is a nonrandomized retrospective study, it is difficult to address these factors. In an attempt to shed light on possible differences in demographics and pre-existing social factors, patients in all groups were analyzed for gender and social factors, such as smoking status, intravenous drug use, and alcohol abuse. There were statistically significant differences (*P* < 0.05) between autograft cohort vs. allograft, autograft/allograft, and BMP-2 cohorts with regards to age and gender. In addition, a statistically significant difference (*P* < 0.05) was found between the illicit drug use status in the autograft/allograft group and remaining cohorts. All other demographics (smoking and alcohol abuse) failed to demonstrate any statistically significant differences.

Nevertheless, our study provides valuable insights into the efficacy of different bone grafting adjuncts in nonunion surgery of long bones, implying that the use of autograft as a grafting adjunct may continue to represent the best practice until higher quality data are available.

## Conclusion

Autograft appears to represent the most efficacious bone grafting adjunct for nonunion surgery of long bones. Time to union, need for revision surgery, and revision bone grafting, as well as new onset or continuing postoperative infection rates were most favorable when autograft was used, compared to allograft, combined autograft/allograft, or rhBMP-2. In light of the growing critical reviews related to the safety and efficacy of rhBMP-2 in other indications, namely in spine surgery, the use of this product should be critically evaluated prior to surgical revision of long bone fracture nonunions.

## Competing interests

The authors declare that they have no competing interests.

## Authors’ contributions

WRS, DJH, and PFS designed the study. MAF, GP, ER, and KI obtained the data. AEW performed the statistical analysis of the data. MAF, WRS, and PFS wrote the manuscript. CM reviewed the manuscript. All authors read and approved the final manuscript.

## References

[B1] Marsh D Concepts of fracture union, delayed union, and nonunion Clin Orthop Relat Res 1998 355 S22 S30 9917623 10.1097/00003086-199810001-00004

[B2] Hak DJ Management of aseptic tibial nonunion J Am Acad Orthop Surg 2011 19 9 563 573 21885702 10.5435/00124635-201109000-00007

[B3] Tzioupis C Giannoudis PV Prevalence of long-bone non-unions Injury 2007 38 Suppl 2 S3 S9 17920415 10.1016/s0020-1383(07)80003-9

[B4] Lynch JR Taitsman LA Barei DP Nork SE Femoral nonunion: risk factors and treatment options J Am Acad Orthop Surg 2008 16 2 88 97 18252839 10.5435/00124635-200802000-00006

[B5] Kanakaris NK Giannoudis PV The health economics of the treatment of long-bone non-unions Injury 2007 38 Suppl 2 S77 S84 17920421 10.1016/s0020-1383(07)80012-x

[B6] Oakley MJ Smith WR Morgan SJ Ziran NM Ziran BH Repetitive posterior iliac crest autograft harvest resulting in an unstable pelvic fracture and infected nonunion: case report and review of the literature Patient Saf Surg 2007 1 1 6 10.1186/1754-9493-1-6 18271999 PMC2241775

[B7] Newman JT Stahel PF Smith WR Resende GV Hak DJ Morgan SJ A new minimally invasive technique for large volume bone graft harvest for treatment of fracture nonunions Orthopedics 2008 31 3 257 261 10.3928/01477447-20080301-29 18351046

[B8] Dimitriou R Mataliotakis GI Angoules AG Kanakaris NK Giannoudis PV Complications following autologous bone graft harvesting from the iliac crest and using the RIA: a systematic review Injury 2011 42 Suppl. 2 S3 S15 21704997 10.1016/j.injury.2011.06.015

[B9] Herscovici D Jr Scaduto JM Use of reamer-irrigator-aspirator technique to obtain autograft for ankle and hindfoot arthrodesis J Bone Joint Surg Br 2012 94 1 75 79 22219251 10.1302/0301-620X.94B1.27690

[B10] Sen MK Micalu T Autologous iliac crest bone graft: should it still be the gold standard for treating nonunions? Injury 2007 38 Suppl. 1 S75 S80 17383488 10.1016/j.injury.2007.02.012

[B11] Kanakaris NK Paliobeis C Nlanidakis N Giannoudis PV Biological enhancement of tibial diaphyseal aseptic non-unions: the efficacy of autologous bone grafting, BMPs and reaming by-products Injury 2007 38 Suppl 2 S65 S75 17920420 10.1016/s0020-1383(07)80011-8

[B12] Giannoudis PV Dinopoulos HT BMPs: options, indications, and effectiveness J Orthop Trauma 2010 24 Suppl 1 S9 S16 20182245 10.1097/BOT.0b013e3181cde5be

[B13] Garrison KR Shemilt I Donell S Ryder JJ Mugford M Harvey I Song F Alt V Bone morphogenetic protein (BMP) for fracture healing in adults Cochrane Database Syst Rev 2010 6 CD006950 10.1002/14651858.CD006950.pub2 PMC6669254 20556771

[B14] Burks MV Nair L Long-term effects of bone morphogenetic protein-based treatments in humans J Long Term Eff Med Implants 2010 20 4 277 293 10.1615/JLongTermEffMedImplants.v20.i4.30 21488821

[B15] Argintar E Edwards S Delahay J Bone morphogenetic proteins in orthopaedic trauma surgery Injury 2011 42 8 730 734 10.1016/j.injury.2010.11.016 21145058

[B16] Carragee EJ Hurwitz EL Weiner BK A critical review of recombinant human bone morphogenetic protein-2 trials in spinal surgery: emerging safety concerns and lessons learned Spine J 2011 11 6 471 491 10.1016/j.spinee.2011.04.023 21729796

[B17] Carragee EJGAJ Weiner BK Rothman DJ Bono CM A challenge to integrity in spine publications: years of living dangerously with the promotion of bone growth factors Spine J 2011 11 6 463 468 10.1016/j.spinee.2011.06.001 21729794

[B18] Mroz TE Wang JC Hashimoto R Norvell DC Complications related to osteobiologics use in spine surgery: a systematic review Spine (Phila Pa 1976) 2010 35 9 S86 S104 20407355 10.1097/BRS.0b013e3181d81ef2

[B19] Frolke JP Patka P Definition and classification of fracture non-unions Injury 2007 38 Suppl 2 S19 S22 17920413 10.1016/s0020-1383(07)80005-2

[B20] Corrales LA Morshed S Bhandari M Miclau T 3rd Variability in the assessment of fracture-healing in orthopaedic trauma studies J Bone Joint Surg Am 2008 90 9 1862 1868 10.2106/JBJS.G.01580 18762645 PMC2663323

[B21] Morshed S Corrales L Genant H Miclau T 3rd Outcome assessment in clinical trials of fracture-healing J Bone Joint Surg Am 2008 90 Suppl 1 62 67 18292359 10.2106/JBJS.G.01556

[B22] Giannoudis PV Dinopoulos HT Autologous bone graft: when shall we add growth factors? Foot Ankle Clin 2010 15 4 597 609 10.1016/j.fcl.2010.09.005 21056859

[B23] Marsell R Einhorn TA Emerging bone healing therapies J Orthop Trauma 2010 24 Suppl 1 S4 S8 20182234 10.1097/BOT.0b013e3181ca3fab

[B24] Govender S Csimma C Genant HK Valentin-Opran A Amit Y Arbel R Aro H Atar D Bishay M Borner MG Chiron P Choong P Cinats J Courtenay B Feibel R Geulette B Gravel C Haas N Raschke M Hammacher E van der Velde D Hardy P Holt M Josten C Ketterl RL Lindeque B Lob G Mathevon H McCoy G Marsh D Recombinant human bone morphogenetic protein-2 for treatment of open tibial fractures: a prospective, controlled, randomized study of four hundred and fifty patients J Bone Joint Surg Am 2002 84-A 12 2123 2134 12473698 10.2106/00004623-200212000-00001

[B25] Jones AL Bucholz RW Bosse MJ Mirza SK Lyon TR Webb LX Pollak AN Golden JD Valentin-Opran A Recombinant human BMP-2 and allograft compared with autogenous bone graft for reconstruction of diaphyseal tibial fractures with cortical defects: a randomized, controlled trial J Bone Joint Surg Am 2006 88 7 1431 1441 10.2106/JBJS.E.00381 16818967

[B26] Aro HT Govender S Patel AD Hernigou P Perera De Gregorio A Popescu GI Golden JD Christensen J Valentin A Recombinant human bone morphogenetic protein-2: a randomized trial in open tibial fractures treated with reamed nail fixation J Bone Joint Surg Am 2011 93 9 801 808 10.2106/JBJS.I.01763 21454742

[B27] Tressler MA Richards JE Sofianos D Comrie FK Kregor PJ Obremskey WT Bone morphogenetic protein-2 compared to autologous iliac crest bone graft in the treatment of long bone nonunion Orthopedics 2011 34 12 e877 e884 22146205 10.3928/01477447-20111021-09

[B28] Weiss KR Cooper GM Jadlowiec JA McGough RL 3rd Huard J VEGF and BMP expression in mouse osteosarcoma cells Clin Orthop Relat Res 2006 450 111 117 16906080 10.1097/01.blo.0000229333.98781.56

[B29] Yoshikawa H Nakase T Myoui A Ueda T Bone morphogenetic proteins in bone tumors J Orthop Sci 2004 9 3 334 340 10.1007/s00776-004-0764-9 15168194

[B30] Krause F Younger A Weber M Recombinant human BMP-2 and allograft compared with autogenous bone graft for reconstruction of diaphyseal tibial fractures with cortical defects J Bone Joint Surg Am 2008 90 5 1168 1169 18451418

[B31] Ritting AW Weber EW Lee MC Exaggerated inflammatory response and bony resorption from BMP-2 use in a pediatric forearm nonunion J Hand Surg [Am] 2012 37 2 316 321 10.1016/j.jhsa.2011.10.007 22119603

[B32] Kinney RC Ziran BH Hirshorn K Schlatterer D Ganey T Demineralized bone matrix for fracture healing: fact or fiction? J Orthop Trauma 2010 24 Suppl 1 S52 S55 20182237 10.1097/BOT.0b013e3181d07ffa

[B33] Takikawa S Bauer TW Kambic H Togawa D Comparative evaluation of the osteoinductivity of two formulations of human demineralized bone matrix J Biomed Mater Res A 2003 65 1 37 42 12635152 10.1002/jbm.a.10345

[B34] Boyce T Edwards J Scarborough N Allograft bone: the influence of processing on safety and performance Orthop Clin North Am 1999 30 4 571 581 10.1016/S0030-5898(05)70110-3 10471762

[B35] Bae HW Zhao L Kanim LE Wong P Delamarter RB Dawson EG Intervariability and intravariability of bone morphogenetic proteins in commercially available demineralized bone matrix products Spine (Phila Pa 1976) 2006 31 12 1299 1306 discussion 1307–1298 10.1097/01.brs.0000218581.92992.b7 16721289

[B36] Bae H Zhao L Zhu D Kanim LE Wang JC Delamarter RB Variability across ten production lots of a single demineralized bone matrix product J Bone Joint Surg Am 2010 92 2 427 435 10.2106/JBJS.H.01400 20124070

[B37] Pape HC Evans A Kobbe P Autologous bone graft: properties and techniques J Orthop Trauma 2010 24 Suppl 1 S36 S40 20182233 10.1097/BOT.0b013e3181cec4a1

[B38] Marino JT Ziran BH Use of solid and cancellous autologous bone graft for fractures and nonunions Orthop Clin North Am 2010 41 1 15 26 10.1016/j.ocl.2009.08.003 19931049

[B39] Ahlmann E Patzakis M Roidis N Shepherd L Holtom P Comparison of anterior and posterior iliac crest bone grafts in terms of harvest-site morbidity and functional outcomes J Bone Joint Surg Am 2002 84-A 5 716 720 12004011 10.2106/00004623-200205000-00003

[B40] DeOrio JK Farber DC Morbidity associated with anterior iliac crest bone grafting in foot and ankle surgery Foot Ankle Int 2005 26 2 147 151 15737257 10.1177/107110070502600206

[B41] Belthur MV Conway JD Jindal G Ranade A Herzenberg JE Bone graft harvest using a new intramedullary system Clin Orthop Relat Res 2008 466 12 2973 2980 10.1007/s11999-008-0538-3 18841433 PMC2628246

[B42] Conway JD Autograft and nonunions: morbidity with intramedullary bone graft versus iliac crest bone graft Orthop Clin North Am 2010 41 1 75 84 10.1016/j.ocl.2009.07.006 19931055

[B43] Stannard JP Sathy AK Moeinpour F Stewart RL Volgas DA Quantitative analysis of growth factors from a second filter using the Reamer-Irrigator-Aspirator system: description of a novel technique Orthop Clin North Am 2010 41 1 95 98 10.1016/j.ocl.2009.07.002 19931057

[B44] Hak DJ Pittman JL Biological rationale for the intramedullary canal as a source of autograft material Orthop Clin North Am 2010 41 1 57 61 10.1016/j.ocl.2009.07.005 19931053

